# High quality draft genome sequence of *Olivibacter sitiensis* type strain (AW-6^T^), a diphenol degrader with genes involved in the catechol pathway

**DOI:** 10.4056/sigs.5088950

**Published:** 2014-03-20

**Authors:** Spyridon Ntougias, Alla Lapidus, James Han, Konstantinos Mavromatis, Amrita Pati, Amy Chen, Hans-Peter Klenk, Tanja Woyke, Constantinos Fasseas, Nikos C. Kyrpides, Georgios I. Zervakis

**Affiliations:** 1 Democritus University of Thrace, Department of Environmental Engineering, Laboratory of Wastewater Management and Treatment Technologies, Xanthi, Greece; 2 Department of Energy Joint Genome Institute, Genome Biology Program, Walnut Creek, CA, USA; 3 Biological Data Management and Technology Center, Lawrence Berkeley National Laboratory, Berkeley, California, USA; 4 Leibniz Institute DSMZ – German Collection of Microorganisms and Cell Cultures, Braunschweig, Germany; 5 Agricultural University of Athens, Electron Microscopy Laboratory, Athens, Greece; 6 King Abdulaziz University, Jeddah, Saudi Arabia; 7 Agricultural University of Athens, Laboratory of General and Agricultural Microbiology, Athens, Greece

**Keywords:** alkaline two-phase olive mill waste, *Bacteroidetes*, *Sphingobacteriaceae*, hemicellulose degradation, *β*-1,4-xylanase, *β*-1,4-xylosidase

## Abstract

*Olivibacter sitiensis* Ntougias et al. 2007 is a member of the family *Sphingobacteriaceae*, phylum *Bacteroidetes*. Members of the genus *Olivibacter* are phylogenetically diverse and of significant interest. They occur in diverse habitats, such as rhizosphere and contaminated soils, viscous wastes, composts, biofilter clean-up facilities on contaminated sites and cave environments, and they are involved in the degradation of complex and toxic compounds. Here we describe the features of *O. sitiensis* AW-6^T^, together with the permanent-draft genome sequence and annotation. The organism was sequenced under the Genomic Encyclopedia for *Bacteria* and *Archaea* (GEBA) project at the DOE Joint Genome Institute and is the first genome sequence of a species within the genus *Olivibacter*. The genome is 5,053,571 bp long and is comprised of 110 scaffolds with an average GC content of 44.61%. Of the 4,565 genes predicted, 4,501 were protein-coding genes and 64 were RNA genes. Most protein-coding genes (68.52%) were assigned to a putative function. The identification of 2-keto-4-pentenoate hydratase/2-oxohepta-3-ene-1,7-dioic acid hydratase-coding genes indicates involvement of this organism in the catechol catabolic pathway. In addition, genes encoding for *β*-1,4-xylanases and *β*-1,4-xylosidases reveal the xylanolytic action of *O. sitiensis*.

## Introduction

The genus *Olivibacter* currently contains six species with validly published names, all of which are aerobic and heterotrophic, non-motile, rod-shaped Gram-negative bacteria [1-3]. Strain AW-6^T^ (= DSM 17696^T^ = CECT 7133^T^ = CIP 109529^T^) is the type strain of *Olivibacter sitiensis* [1], which is the type species of the genus *Olivibacter*. The strain was isolated from alkaline alperujo, an olive mill sludge-like waste produced by two-phase centrifugal decanters located in the vicinity of Toplou Monastery, Sitia, Greece [1]. The genus name derived from the Latin term *oliva* and the Neo-Latin *bacter*, meaning a rod-shaped bacterium living in olives/olive processing by-products [1]. The Neo-Latin species epithet *sitiensis* pertains to the region Sitia (Crete, Greece) where the olive mill is operating [1]. The other species of the genus are *O. soli*, *O. ginsengisoli*, *O. terrae*, *O. oleidegradans* and *O. jilunii* [2-4]. *O. soli* and *O. ginsengisoli* were isolated from soil of a ginseng field [2], *O. terrae* from a compost prepared of cow manure and rice straw [2], *O. oleidegradans* from a biofilter clean-up facility in a hydrocarbon-contaminated site [3] and *O. jilunii* from a DDT-contaminated soil [4]. *O. sitiensis* can be distinguished from *O. soli*, *O. ginsengisoli* and *O. terrae* on the basis of temperature and NaCl concentration ranges for growth, in its ability to assimilate N-acetyl-D-glucosamine, L-histidine, maltose and sorbitol, and for expression of naphthol-AS-BI-phosphohydrolase, in the presence/absence of iso-C_15: 1_ F, C_16: 1_ 2-OH, anteiso-C_17: 1_ B and/or iso-C_17: 1_ I, and in by its DNA G+C content [1,2,4]. Moreover, it differs from *O. soli* in terms of L-arabinose assimilation and valine arylamidase expression, from *O. ginsengisoli* in terms of inositol, mannitol and salicin assimilation and in oxidase reaction test, and from *O. terrae* in terms of L-arabinose and mannitol assimilation, and *β*-glucuronidase and valine arylamidase expression [1,2,4]. *O. sitiensis* can be differentiated from *O. oleidegradans* on the basis of DNA G+C content, pH upper limit for growth, in the ability for assimilation of D-adonitol, L-arabinose, N-acetyl-D-glucosamine, L-histidine, D-lyxose, maltose, melezitoze, salicin and turanose, and for expression of esterase, *β*-galactosidase, *α*-mannosidase, urease and valine arylamidase as well as in the presence/absence of some minor fatty acid components of membrane lipids, menaquinone-6 (as minor respiratory quinone) and aminophospholipids (as cellular polar lipids) [1,3,4]. In addition, *O. sitiensis* can be distinguished from *O. jelunii* on the basis of DNA G+C content, pH, temperature and NaCl concentration upper limits for growth, lactose fermentation, in the ability for assimilation of acetate, L-arabinose, N-acetyl-D-glucosamine, L-histidine, malonate, maltose, D-mannose, salicin and L-serine, and for expression of *α*-mannosidase, oxidase and valine arylamidase as well as in the presence/absence of some minor fatty acid components of membrane lipids, menaquinone-8 (as minor respiratory quinone) and aminophospholipids (as cellular polar lipids) [1,4]. Here we present a summary classification and a set of features for *O. sitiensis* AW-6^T^, together with the description of the permanent-draft genome sequencing and annotation.

## Classification and features

The 16S rRNA gene sequence of *O. sitiensis* AW-6^T^ was compared using NCBI BLAST under default settings (e.g., considering only the high-scoring segment pairs (HSPs) from the best 250 hits) with the most recent release of the Greengenes database [5] and the relative frequencies of taxa and keywords (reduced to their stem [6]) were determined and weighted by BLAST scores. The frequency of genera that belonged to the family *Sphingobacteriaceae* was 61.8%. The most frequently occurring genera were in order *Sphingobacterium* (27.7%), *Pedobacter* (17.1%), *Flavobacterium* (8.5%), *Olivibacter* (6.4%), *Hymenobacter* (6.4%), *Mucilaginibacter* (4.3%), *Cytophaga* (4.3%), *Flectobacillus* (4.3%), *Parapedobacter* (2.1%), *Pseudosphingobacterium* (2.1%) and ‘*Hevizibacter*’ (2.1%) (47 hits in total). The 16S rRNA gene sequence of *O. sitiensis* AW-6^T^ was the only hit on members of the species in INSDC (=EMBL/NCBI/DDBJ) under the accession number DQ421387 (=NR_043805). Among all other species, the two yielding the highest score were *Parapedobacter koreensis* Jip14^T^ (DQ680836) [7] and *Olivibacter ginsengisoli* Gsoil 060^T^ (AB267716) [2], showing similarity in 16S rRNA gene of 90.1% (both of them) and HSP coverages of 99.8% and 99.9% respectively. It is noteworthy that the Greengenes database uses the INSDC (=EMBL/NCBI/DDBJ) annotation, which is not an authoritative source for nomenclature or classification. The highest-scoring environmental sequences was AM114441 ['Interactions U(VI) added natural dependence on various incubation conditions soil uranium mining waste pile clone JG35+U2A-AG9'], which showed identity of 90.3% with HSP coverage of 86.1%. The most frequently occurring keywords within the labels of all environmental samples that yielded hits were 'rumen' (23.1%), 'oil' (10.8%), 'water' (9.7%), 'soil' (9.7%), 'fluid' (9.1%) and 'gut' (9.1%) (186 hits in total). The most frequently occurring keywords within the labels of those environmental samples that yielded hits of a higher score than the highest scoring species were 'waste' (50.0%) and 'soil' (50.0%) (4 hits in total), which are keywords with biological meaning fitting the environment from which *O. sitiensis* AW-6^T^ was isolated.

**Figure 1:**
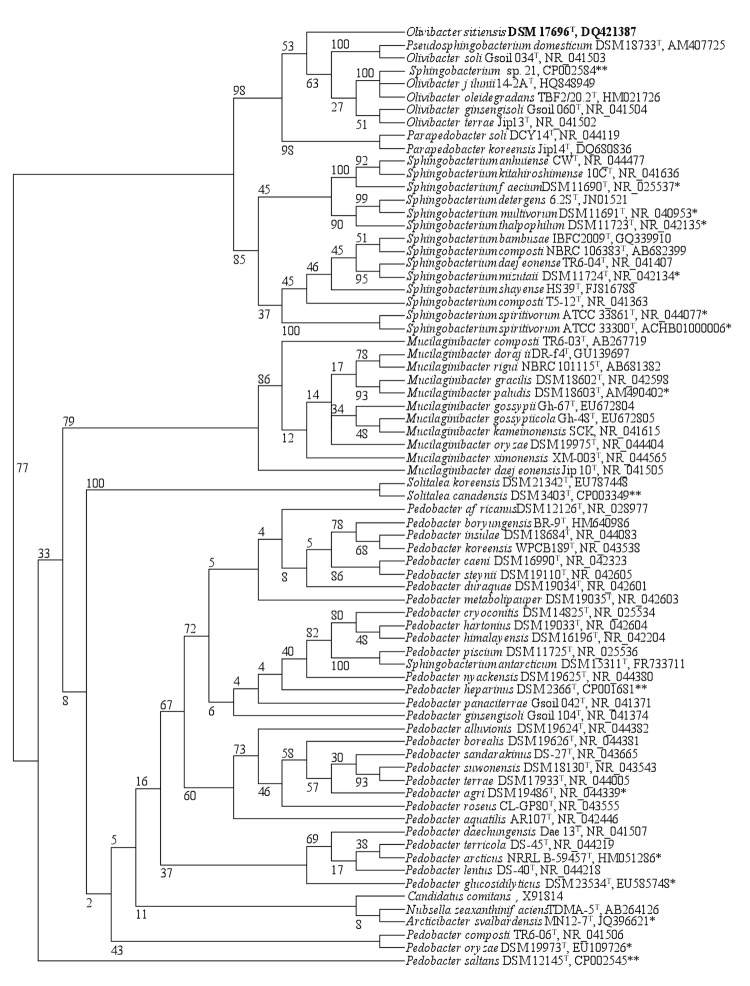
Phylogenetic trees highlighting the position of *O. sitiensis* relative to the type strains of the species within the family *Sphingobacteriaceae*. The tree was inferred from 1,288 aligned characters [8,9] of the 16S rRNA gene sequence under A) the maximum likelihood (ML) [10] and B) the maximum-parsimony criterion. In ML tree, the branches are scaled in terms of the expected number of substitutions per site. Numbers adjacent to the branches are support values from 100 ML bootstrap replicates (A) and from 1,000 maximum-parsimony bootstrap replicates (B) [11]. Lineages with strain genome sequencing projects registered in GOLD [12] are labeled with one asterisk, while those listed as 'Complete and Published' with two asterisks (e.g. *Pedobacter heparinus* [13] and *P. saltans* [14]).

Figure 1 shows the phylogenetic neighborhood of *O. sitiensis* in the 16S rRNA gene sequence-based trees constructed. Independently from the clustering method applied, all *Olivibacter* species together with *Pseudosphingobacterium domesticum* and ‘*Sphingobacterium*’ sp. 21 fell into a distinct cluster, indicating the unique phylogenetic position of genus *Olivibacter* and the necessity for reconsidering the taxonomic status of the genus *Pseudosphingobacterium*. In addition, ‘*Sphingobacterium*’ sp. 21 should be assigned to the genus *Olivibacter*, and not to the genus *Sphingobacterium*. In the ML tree, members of the genus *Parapedobacter* branched together with *O. sitiensis*, although the unique topology of the genus was established by applying a character-based (parsimony) method. As previously stated by Ntougias et al. [1], *S. antarcticum* should be reassigned to the genus *Pedobacter*.

**Figure 2:**
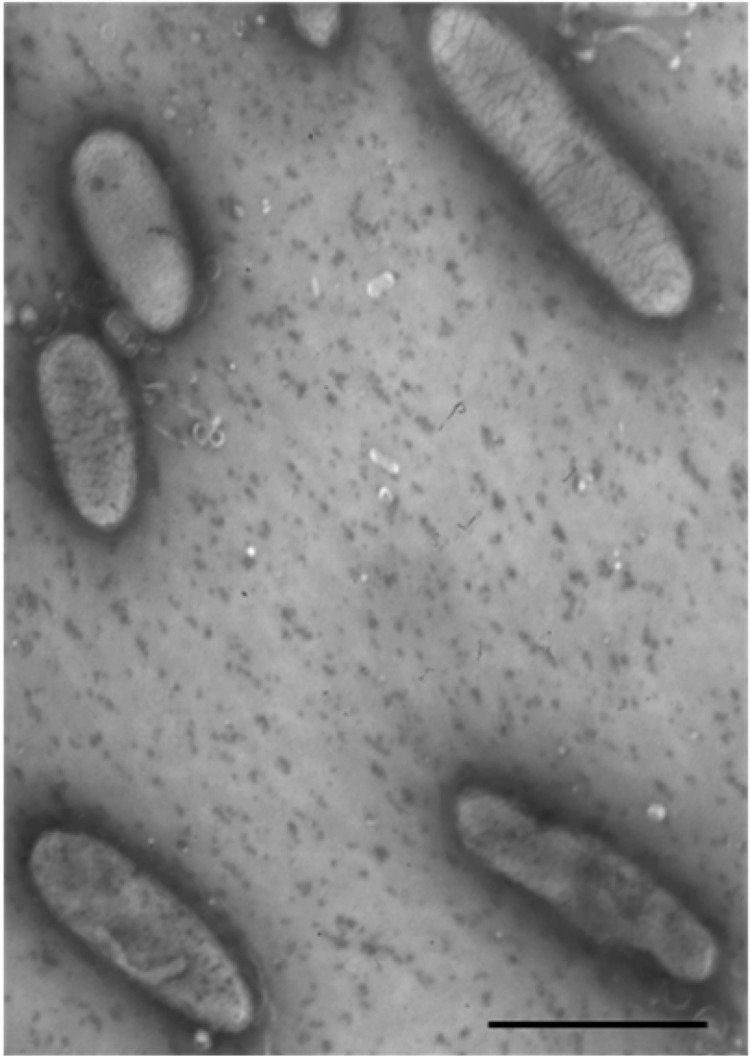
Electron micrograph of *O. sitiensis* AW-6^T^ negatively-stained cells. Bar represents 1 μm.

Cells of *O. sitiensis* AW-6^T^ are Gram-negative non-motile rods [1] with a length of 1.0-1.3 μm and a width of 0.2-0.3 μm (Table 1 and Figure 2). The temperature range for growth is 5-45°C, with an optimum at 28–32°C [1]. *O. sitiensis* is neutrophilic, showing no growth at 30 g L^-1^ NaCl [1]. The pH for growth ranges between 5 and 8, with pH values of 6-7 being the optimum [1]. *O. sitiensis* is strictly aerobic and chemo-organotrophic; it assimilates mostly D(+)-glucose, protocatechuate and D(+)-xylose, while L-cysteine, D(-)-fructose, D(+)-galactose, L-histidine, lactose, sorbitol and sucrose are also utilized by strain AW-6^T^ [1]. *O. sitiensis* was found to be sensitive to ampicillin, bacitracin, chloramphenicol, penicillin, rifampicin, tetracycline and trimethoprim, and resistant to kanamycin, polymixin B and streptomycin (antibiotics’ concentration of 50 μg ml^-1^) [1].

**Table 1:** Classification and general features of *O. sitiensis* AW-6^T^, according to the MIGS recommendations [15].

**MIGS ID**	**Property**	**Term**	**Evidence code**
		Domain *Bacteria*	TAS [16]
		Phylum *Bacteroidetes*	TAS [17,18]
		Class *Sphingobacteriia*	TAS [18,19]
	Current classification	Order *Sphingobacteriales*	TAS [18,20]
		Family *Sphingobacteriaceae*	TAS [21]
		Genus *Olivibacter*	TAS [1]
		Species *Olivibacter sitiensis*	TAS [1]
		Type-strain AW-6^T^	TAS [1]
	Gram stain	negative	TAS [1]
	Cell shape	rod	TAS [1]
	Motility	non-motile	TAS [1]
	Sporulation	non-sporulating	TAS [1]
	Temperature range	mesophile, 5-45°C	TAS [1]
	Optimum temperature	28-32°C	TAS [1]
	Salinity	neutrophilic and non-halotolerant - no growth at 30 g l^-1^ NaCl	TAS [1]
MIGS-22	Oxygen requirement	strictly aerobic	TAS [1]
	Carbon source	carbohydrates and amino-acids, utilization of protocatechuate and sorbitol	TAS [1]
	Energy metabolism	chemo-organotroph	TAS [1]
MIGS-6	Habitat	olive mill waste	TAS [1]
MIGS-15	Biotic relationship	free living	TAS [1]
MIGS-14	Pathogenicity	none	NAS
	Biosafety level	1	TAS [22]
MIGS-23.1	Isolation	alkaline two-phase olive mill waste (alkaline alperujo)	TAS [1]
MIGS-4	Geographic location	Toplou Monastery, Sitia, Crete, Greece	TAS [1]
MIGS-5	Sample collection time	year 2003	NAS
MIGS-4.1	Latitude	35.220	TAS [1]
MIGS-4.2	Longitude	26.216	TAS [1]
MIGS-4.3	Depth	surface	NAS
MIGS-4.4	Altitude	161 m	NAS

Evidence codes - TAS: Traceable Author Statement (i.e. a direct report exists in the literature); NAS: Non-traceable Author Statement (i.e. not directly observed for the living, isolated sample, but based on a generally accepted property for the species, or anecdotal evidence). These evidence codes are from the Gene Ontology project [23]. If the evidence code is IDA, then the property was directly observed for a living isolate by one of the authors or an expert mentioned in the acknowledgements.

### Chemotaxonomy

The major polar lipids of *O. sitiensis* are phosphatidylethanolamine (PE), phosphatidylmonomethylethanolamine (PME), phosphatidylinositol mannoside (PIM), an unknown phospholipid (PL) and an unknown non-phosphorylated lipid (UL) [4]. Moreover, the main membrane fatty acids of *O. sitiensis* are C_16: 1_ω7c and/or iso-C_15:0_ 2-OH, iso-C_15:0_, iso-C_17:0_ 3-OH and C_16:0_ [1]. The only respiratory quinone found in *O. sitiensis* is menaquinone, with seven isoprene subunits (MK-7) [1].

## Genome sequencing and annotation

### Genome project history

This microorganism was selected for sequencing on the basis of its phylogenetic position [24,25], and is part of the Genomic Encyclopedia of Type Strains, Phase I: the one thousand microbial genomes (KMG) project [26] which aims in increasing the sequencing coverage of key reference microbial genomes. The genome project is deposited in the Genomes On Line Database [12] and the genome sequence is available from GenBank. Sequencing, finishing and annotation were performed by the DOE Joint Genome Institute (JGI) using state of the art sequencing technology [27]. A summary of the project information is presented in Table 2.

**Table 2:** Genome sequencing project information.

**MIGS ID**	**Property**	**Term**
MIGS-31	Finishing quality	High-Quality Draft
MIGS-29	Sequencing platforms	Illumina
MIGS-31.2	Sequencing coverage	120×
MIGS-30	Assemblers	ALLPATHS v. r41043
MIGS-32	Gene calling method	Prodigal 2.5
	Genbank ID	ATZA00000000
	Genbank Date of Release	September 5, 2013
	GOLD ID	Gi11724
	NCBI project ID	165253
	Database: IMG	2515154027
MIGS-13	Source material identifier	DSM 17696^T^
	Project relevance	GEBA-KMG, Tree of Life, Biodegradation

### Growth conditions and DNA isolation

*O. sitiensis* strain AW-6^T^ was grown aerobically in DSMZ medium 92 (trypticase soy yeast extract medium) [28] at 28°C. DNA was isolated from 0.5-1 g of cell paste using Jetflex Genomic DNA purification kit (Genomed_600100) following the standard protocol as recommended by the manufacturer but applying a modified cell lysis procedure (1 hour incubation at 58°C with additional 50 µl proteinase K followed by overnight incubation on ice with additional 200 µl PPT-buffer). DNA is available via the DNA Bank Network [29].

### Genome sequencing and assembly

The draft genome of *Olivibacter sitiensis* DSM 17696 was generated at the DOE Joint genome Institute (JGI) using the Illumina technology. An Illumina Standard shotgun library was constructed and sequenced using the Illumina HiSeq 2000 platform, which generated 13,155,872 reads totaling 1,973.4 Mbp. All general aspects of library construction and sequencing performed at the JGI can be found at the JGI website [30]. All raw Illumina sequence data were passed through DUK, a filtering program developed at JGI, which removes known Illumina sequencing and library preparation artifacts (Mingkun L, unpublished). The following steps were then performed for assembly: (i) filtered Illumina reads were assembled using Velvet (version 1.1.04) [31], (ii) 1–3 Kbp simulated paired end reads were created from Velvet contigs using wgsim [32] (iii) Illumina reads were assembled with simulated read pairs using Allpaths–LG (version r41043) [33]. The final draft assembly contained 110 contigs in 110 scaffolds. The total size of the genome is 5.1 Mbp and the final assembly is based on 605.8 Mbp of Illumina data, which provides an average 120.0× coverage of the genome.

### Genome annotation

Genes were identified using Prodigal [34] as part of the DOE-JGI Annotation pipeline [35]. The predicted CDSs were translated and used to search the National Center for Biotechnology Information (NCBI) non-redundant database, UniProt, TIGRFam, Pfam, PRIAM, KEGG, COG, and InterPro databases. Additional gene prediction analysis and functional annotation was performed within the Integrated Microbial Genomes (IMG-ER) [36].

## Genome properties

The genome is 5,053,571 bp long and comprises 110 scaffolds with an average GC content of 44.61% (Table 3). Of the 4,565 genes predicted, 4,501 were protein-coding genes and 64 RNA genes. Most protein-coding genes (68.52%) were assigned to a putative function, while the remaining ones were annotated as hypothetical proteins. The distribution of genes into COGs functional categories is presented in Table 4.

**Table 3:** Genome statistics.

**Attribute**	**Value**	**% of Total^a^**
Genome size (bp)	5,053,571	100.00%
DNA coding region (bp)	4,534,282	89.72%
DNA G+C content (bp)	2,254,441	44.61%
DNA scaffolds	110	
Total genes	4,565	
RNA genes	64	1.40%
tRNA genes	47	1.03%
Protein-coding genes	4,501	98.60%
Genes with function prediction (proteins)	3,128	68.52%
Genes in paralog clusters	1,777	38.93%
Genes assigned to COGs	3,062	67.08%
Genes assigned Pfam domains	3,471	76.04%
Genes with signal peptides	501	10.97%
Genes with transmembrane helices	1,124	24.62%
CRISPR repeats	0	

^a^ The total is based on either the size of the genome in base pairs or the total number of protein coding genes in the annotated genome.

**Table 4:** Number of genes associated with the general COG functional categories.

**Code**	**Value**	**%age**	**Description**
J	159	4.7	Translation, ribosomal structure and biogenesis
A	1	0.0	RNA processing and modification
K	283	8.4	Transcription
L	190	5.7	Replication, recombination and repair
B	1	0.0	Chromatin structure and dynamics
D	22	0.6	Cell cycle control, cell division, chromosome partitioning
Y	0	0.0	Nuclear structure
V	99	2.9	Defense mechanisms
T	197	5.9	Signal transduction mechanisms
M	274	8.1	Cell wall/membrane biogenesis
N	7	0.2	Cell motility
Z	0	0.0	Cytoskeleton
W	0	0.0	Extracellular structures
U	63	1.9	Intracellular trafficking and secretion, and vesicular transport
O	120	3.6	Posttranslational modification, protein turnover, chaperones
C	168	5.0	Energy production and conversion
G	259	7.7	Carbohydrate transport and metabolism
E	211	6.3	Amino acid transport and metabolism
F	61	1.8	Nucleotide transport and metabolism
H	148	4.4	Coenzyme transport and metabolism
I	107	3.2	Lipid transport and metabolism
P	238	7.1	Inorganic ion transport and metabolism
Q	52	1.5	Secondary metabolites biosynthesis, transport and catabolism
R	419	12.5	General function prediction only
S	280	8.3	Function unknown
-	1,503	32.9	Not in COGs

Based on genomic analysis of the metabolic features, *O. sitiensis* is an auxotroph for L-alanine, L-arginine, L-histidine, L-isoleucine, L-leucine, L-lysine, L-phenylalanine, L-proline, L-serine, L-tyrosine, L-tryptophan and L-valine, and a prototroph for L-aspartate, L-glutamate and glycine. Selenocysteine and biotin cannot be synthesized by *O. sitiensis*. Strain AW-6^T^ can utilize L-arabinose and maltose (via orthophosphate activation), whereas no maltose hydrolysis is achieved [1].

Genome analysis revealed the genetic and molecular bases of the degradation of recalcitrant compounds by *O. sitiensis*. The ability of *O. sitiensis* to degrade phenolic compounds is verified by the distribution of genes encoding oxidoreductases that act on diphenols and related substances and by the 2-keto-4-pentenoate hydratase/2-oxohepta-3-ene-1,7-dioic acid hydratase-coding genes that are involved in the catechol pathway. Genes encoding *β*-1,4-xylanases and *β*-1,4-xylosidases were also identified in the genome of strain AW-6^T^, indicating that *O. sitiensis* is a xylanolytic bacterium involved in the cleavage of *β*-1,4-xylosic bonds in hemicelluloses. The existence of protocatechuate 3,4-dioxygenase (dioxygenase_C)-coding genes are indicative of the ability of this bacterium to degrade benzoate and 2,4-dichlorobenzoate. Genes encoding carboxymethylenebutenolidase were distributed in the genome of *O. sitiensis*, indicating its potential for hexachlorocyclohexane and 1,4-dichlorobenzene degradation. Oxidoreductases related to aryl-alcohol dehydrogenases were predicted, showing that *O. sitiensis* may be also involved in biphenyl and toluene/xylene degradation. This is also strengthened by the identification of an uncharacterized protein, possibly involved in aromatic compounds catabolism. Moreover, putative multicopper oxidases with possible laccase-like activity were identified. Mercuric reductase- and arsenate reductase-coding genes as well as organic solvent tolerance and chromate transport proteins encoded in the genome indicate possible resistance of *O. sitiensis* to the presence of heavy metals and organic solvents.
